# An Unusual Exostosis of the Scapula Mimicking a Glenoid‐Like Structure in Snapping Scapula Syndrome

**DOI:** 10.1155/cro/1457746

**Published:** 2025-12-18

**Authors:** Alaa Elsenbsy, Jeanni Zbinden, Alexandre Lädermann, Michael Simoni

**Affiliations:** ^1^ Department of Orthopedic and Trauma Surgery, Faculty of Medicine, South Valley University, Qena, Egypt, svu.edu.eg; ^2^ Division of Orthopaedics and Trauma Surgery, Hôpital de La Tour, Meyrin, Switzerland, la-tour.ch; ^3^ Faculty of Medicine, University of Geneva, Geneva 4, Switzerland, unige.ch; ^4^ Division of Orthopaedics and Trauma Surgery, Department of Surgery, Geneva University Hospitals, Geneva 14, Switzerland, hug-ge.ch; ^5^ FORE (Foundation for Research and Teaching in Orthopedics, Sports Medicine, Trauma, and Imaging in the Musculoskeletal System), Meyrin, Switzerland; ^6^ Division of Orthopaedics Hospital Copa Star and IDOR Institute, Rio de Janeiro, Brazil

## Abstract

**Introduction:**

Snapping scapula syndrome is an unusual condition characterized by an audible, grating, and snapping sound upon shoulder movement, often observed in young adults. Clinical severity ranges from gentle friction sounds to louder grating noises or crepitus, which may or may not be associated with pain and limited motion. Multiple etiologies include congenital malformations such as Sprengel′s deformity, exostosis, subscapular mass, and scapulothoracic bursitis.

**Case Presentation:**

A 28‐year‐old right‐handed male presented a 12‐year history of crepitus and snapping of the right scapula, with pain and worsening crepitus in the past year. Clinical examination revealed a symmetrical glenohumeral range of motion with combined scapular dyskinesia. CT scan showed a bony mass near the superior border and superomedial angle of the scapula, resembling a second glenoid with a smaller, less concave surface. The mass included a protuberance that suggested a second coracoid process and spine of the scapula. Surgical excision of the bony mass and surrounding bursa, along with partial resection of the superomedial angle, was performed. The patient fully recovered after 3 months and presented no symptoms of recurrence at the 2‐year follow‐up. The excised mass consisted of mature bony tissue with interspersed trabeculae, suggestive of exostosis.

**Conclusion:**

Bone masses arising from the scapula vary in origin, presentation, and morphology. We describe a unique bony projection resembling a second glenoid, treated successfully with surgical resection and resulting in complete cosmetic and functional recovery.

## 1. Introduction

Snapping scapula syndrome is an unusual condition in which patients, often young adults, present with an audible, grating, and snapping sound upon shoulder movement [1, 2]. Upon clinical examination, the severity of this condition can range from a gentle friction sound to a louder grating noise or crepitus, which may or may not be associated with pain and limited motion. Additionally, the snapping scapula is easily palpable during shoulder movement.

Snapping and winging scapulae have multiple etiologies, including congenital malformations of the scapula that vary from nearly complete absence to abnormal scapular shape and position, such as Sprengel’s deformity, exostosis, subscapular mass, and scapulothoracic bursitis [3–5].

Masses arising from the scapula can range from benign tumors like osteochondroma, exostosis, and elastofibroma to malignant tumors such as osteosarcoma, Ewing tumor, and chondrosarcoma [6–8]. Osteochondroma is the most common type of benign tumor arising from the surface of a bone, commonly the metaphysis of long bones, and relatively rare in flat bones like the scapula [9, 10]. However, the most common benign tumor of the scapula is osteochondroma, which usually arises from the ventral surface, occasionally from the dorsal surface, and rarely from the superomedial angle [11–13]. Approximately 20%–25% of patients with Sprengel′s shoulder deformity have an omovertebral connection [14]. This connection can be either fibrous or osseous and is located between the superomedial border of the scapula and the spinous process, transverse process, or the lamina of the C4–7 cervical vertebrae [15].

The goal of this report is to describe an unusual exostosis of the scapula mimicking a glenoid‐like structure in snapping scapula syndrome.

## 2. Case Presentation

A 28‐year‐old right‐handed male presented crepitus and snapping of his right scapula for 12 years. Pain and progressively worsening crepitus appeared the previous year. He had no history of treatment. He is graduated in business administration. He regularly works out and plays footvolley.

Clinical examination revealed a symmetrical glenohumeral range of motion with combined scapular dyskinesia. Computed tomography (CT) scan of the right shoulder with reconstruction showed a bony mass close to the superior border and superomedial angle of the scapula, resembling a second glenoid with a smaller, less concave surface. In addition, a protuberance emerging from the base was likely a second coracoid process and spine of the scapula (Figure 1).

Figure 1(a) 3D reconstruction CT scan and (b) axial CT view of the right shoulder shows a bony mass at the superior border of the right scapula with a protuberance at its base (white arrows). Reproduced from http://www.BeeMed.com, with permission.

**Figure:**
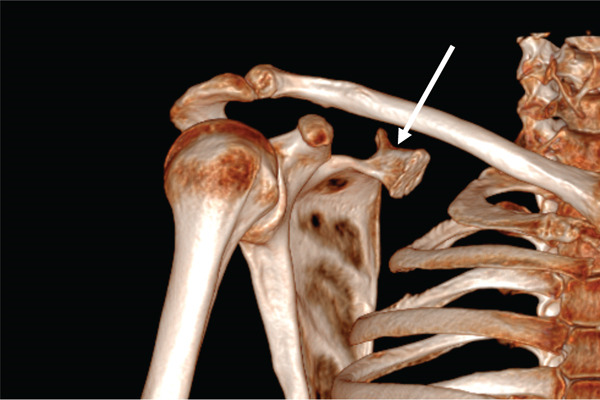
(a)

**Figure:**
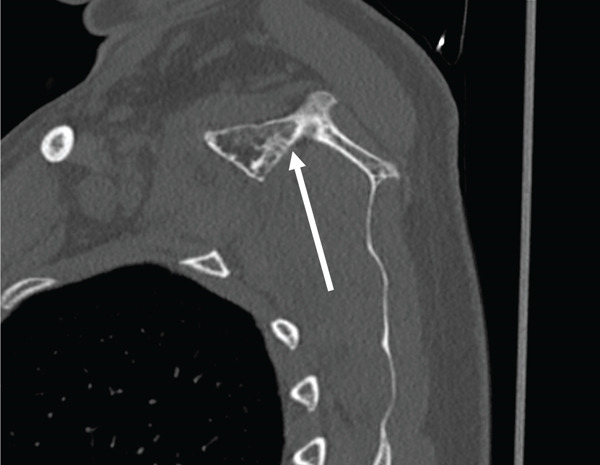
(b)

Shoulder physiotherapy was recommended to strengthen the scapular stabilizers. Crepitus persisted despite pain improvement. To eliminate the remaining symptoms, the patient consented to surgical excision of the bony mass and the surrounding bursa with partial resection of the superomedial angle.

### 2.1. Ethics and Consent Statement

Written informed consent was obtained from the patient for publication of this case report and accompanying images. Institutional review board approval was not required for this type of study.

### 2.2. Surgical Technique

**Figure 2:**
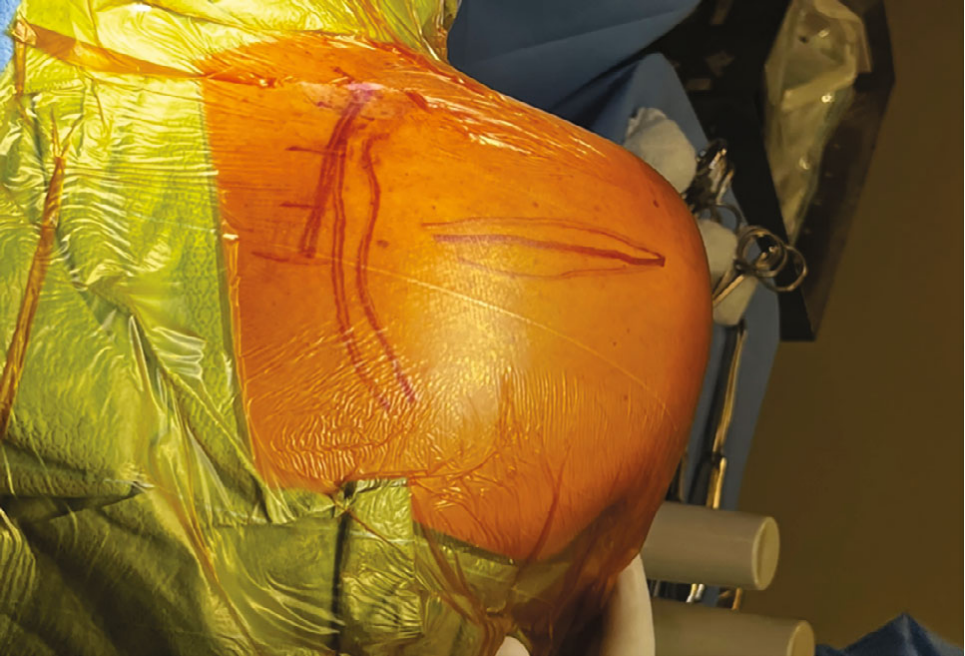
The perioperative photo shows lateral positioning with drawn anatomical landmarks. The vertical incision is located along the medial border of the scapula. Reproduced from http://www.BeeMed.com, with permission.

**Figure 3:**
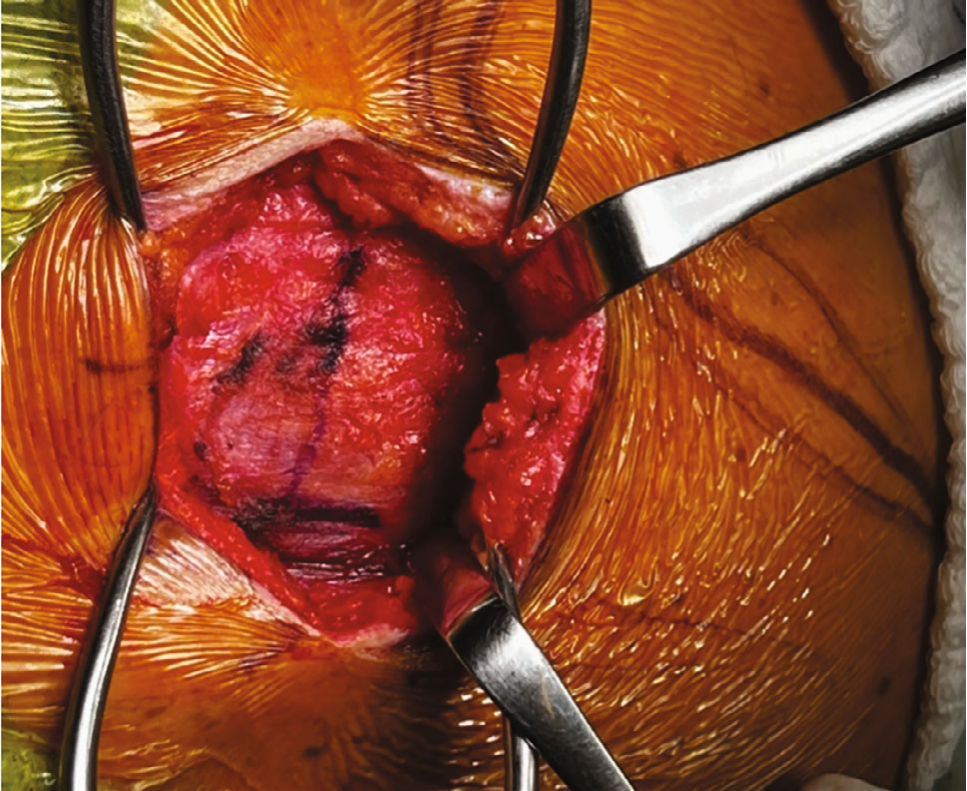
A line is drawn over the trapezius from the scapular spine to expose the superior angle of the scapula. Reproduced from http://www.BeeMed.com, with permission.

The patient was positioned in lateral decubitus over his contralateral arm with his arm crossing at 80° of forward flexion. An 8‐cm vertical incision parallel to the medial border of the upper scapula was performed (Figure 2). The medial scapula was marked, and a transverse incision in the trapezius revealed a well‐divided space over the bone (Figure 3). Two retractors were used. After a subperiosteal dissection of the aponeurosis from the supraspinatus and rhomboids, and a small triangle of the superior medial angle of the scapula, the exostosis was clearly identified. Using an osteotome, the lesion was resected from the scapula together with the extensive adjacent anterior bursal tissue. The dimension of the excised mass was 3 cm by 1.5 cm (Figure 4a,b). For cosmetic purposes, the closure respected all layers, including the aponeurosis, trapezius, subcutaneous, and subcuticular skin. The patient was postoperatively immobilized in a sling.

Figure 4(a) The mass is dissected and exposed intraoperatively. (b, c) The mass is measured postoperatively. Reproduced from http://www.BeeMed.com, with permission.

**Figure:**
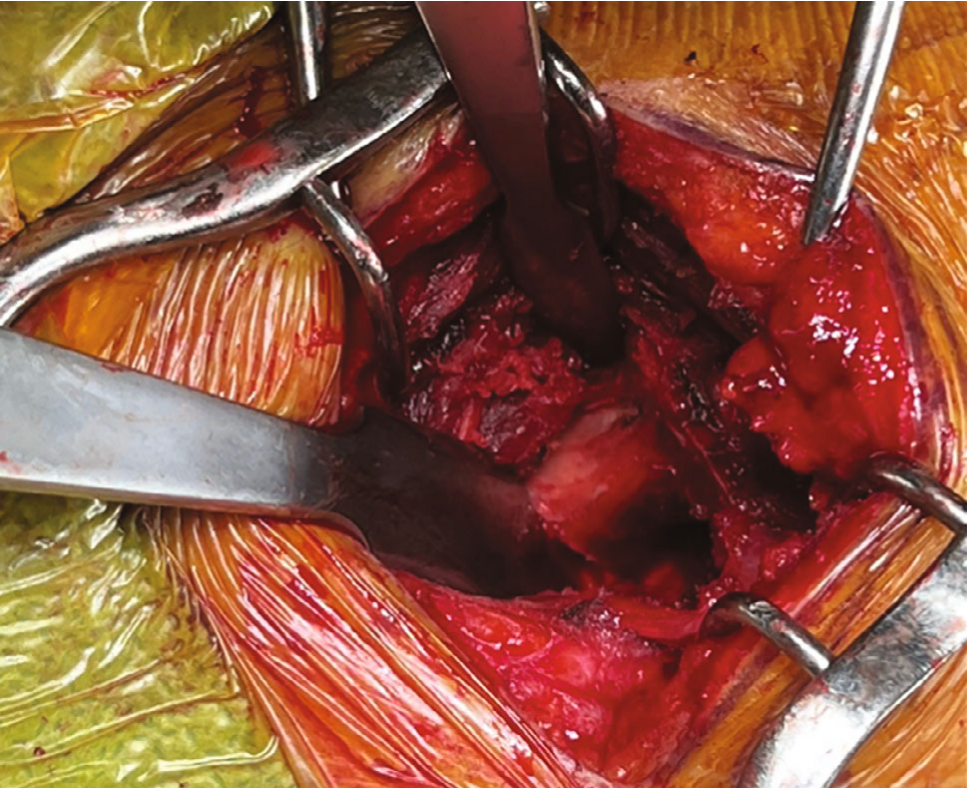
(a)

**Figure:**
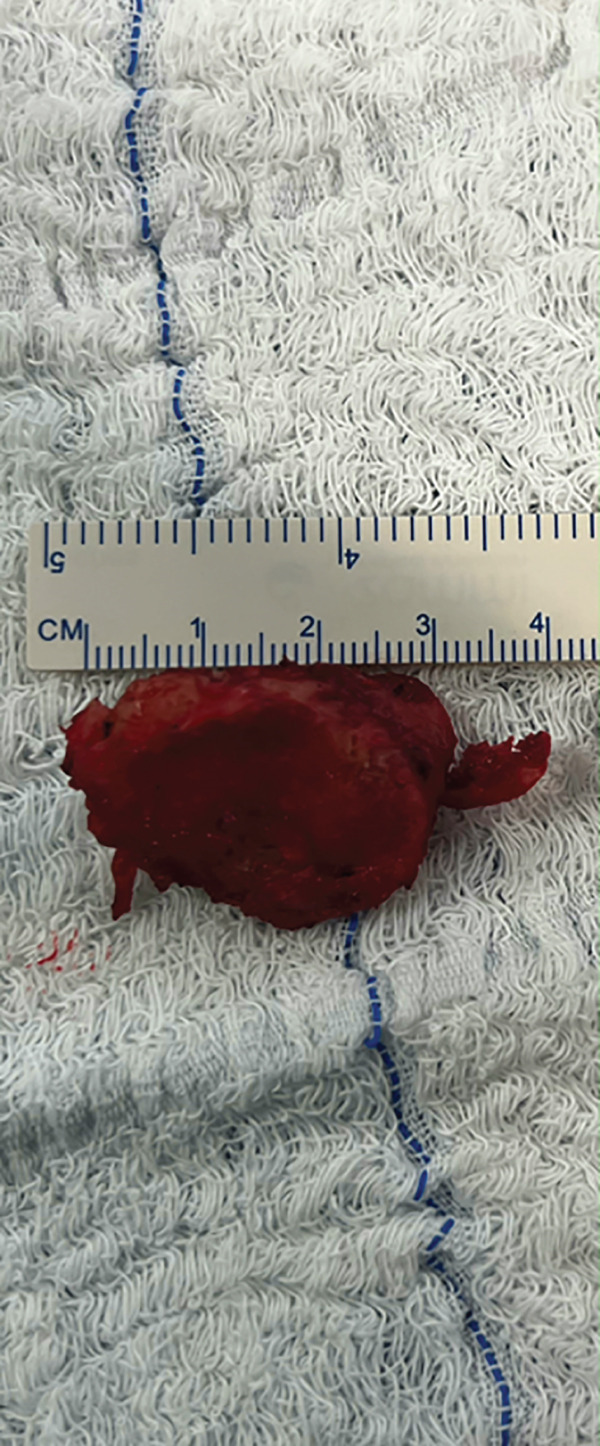
(b)

**Figure:**
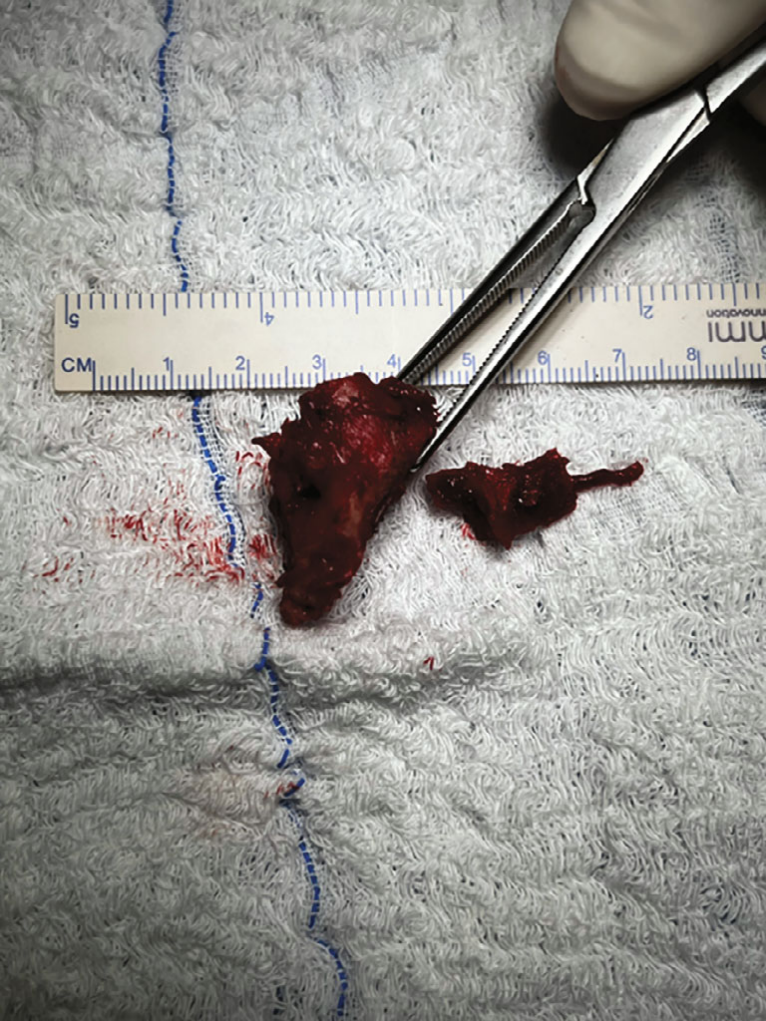
(c)

### 2.3. Postoperative Rehabilitation and Course

**Figure 5:**
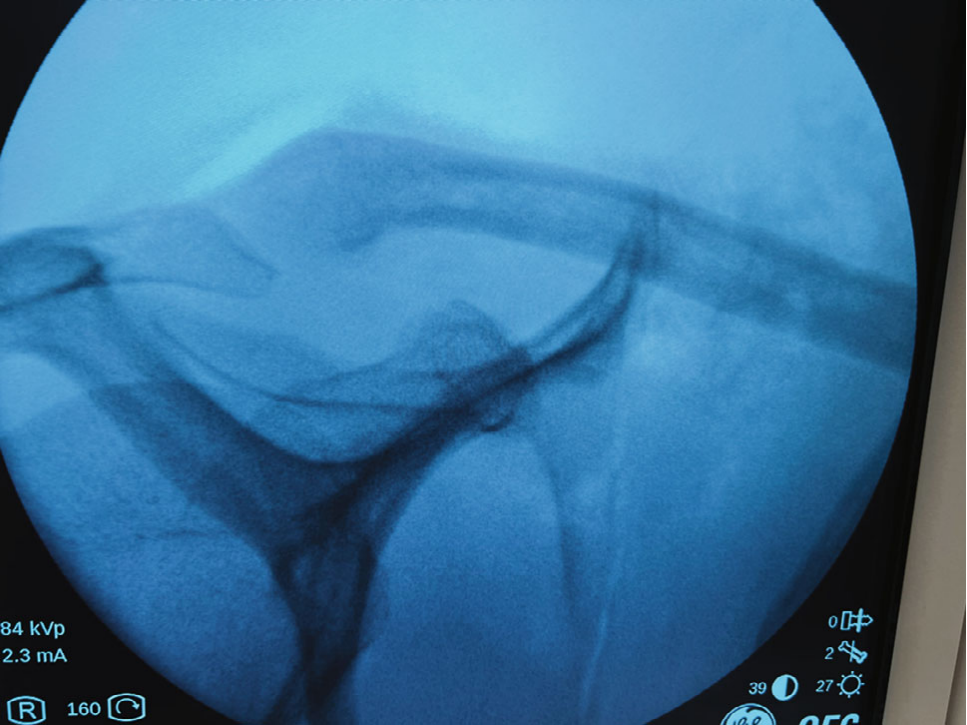
Postoperative anteroposterior view reveals a right shoulder X‐ray after excision of the bony exostosis. Reproduced from http://www.BeeMed.com, with permission.

A sling was used for approximately 4 weeks to respect muscle healing. A Phase 1 exercise, including elbow flexion, cervical motion, shoulder external rotation, and small “walking on the thigh,” was recommended starting the day after surgery. Within 2 weeks, pendulum exercises were added. After 4 weeks, active motion and rehabilitation was proposed. Strengthening exercises, however, were only permitted after 2 months. After 3 months, the patient was to resume regular athletic activity. After 2 years, at the final follow‐up, the patient presented a satisfactory outcome, with no symptoms of recurrence (Figure 5).

### 2.4. Histopathological Examination

Histopathological examination showed mature lamellar bone with trabecular architecture, fatty and hematopoietic marrow, surrounded by fibrous connective tissue. No cartilage cap was identified, ruling out osteochondroma.

## 3. Discussion

Previous literature concerning periscapular masses has described various etiologies, including tumors originating from the scapula (such as osteochondroma), benign and malignant soft tissue masses from surrounding muscles, and congenital anomalies like omovertebral connections [7, 16–19]. Masses in this region are unusual in origin, presentation, and character. A bony lesion on the anterior aspect of the scapula or the posterior aspect of the thoracic cage, particularly a subscapular mass, may lead to snapping scapula syndrome [15].

Osteochondroma is the most common benign bone tumor and a well‐recognized cause of snapping scapula. It typically presents as a cartilage‐capped bony projection arising from the bone surface, most often from the metaphysis of long bones, but can occur in the scapula. In the scapular region, it is usually located on the ventral surface, where it can cause crepitus, pain, and mechanical impingement with the thoracic cage [20–22]. Several authors, including Milch and Parsons, have reported scapular winging and snapping caused by osteochondroma, with symptom resolution after surgical excision [1, 23]. Hollingshead and James described a locking scapula caused by an osteochondroma trapped between the ribs [24].

In the present case, histopathology demonstrated mature lamellar bone with fatty and hematopoietic marrow, surrounded by fibrous connective tissue, and no cartilage cap—features more consistent with an exostosis than with an osteochondroma. Nonetheless, the absence of a cartilage cap in histology could be due to complete ossification of a preexisting osteochondroma, a finding occasionally reported in older patients.

The differential diagnosis of bony scapular lesions includes osteochondroma, characterized by a cartilage cap (usually greater than 2 mm in adults and up to 3 cm in skeletally immature patients), continuity of cortical and medullary bone with the host bone, and frequent presentation in younger patients. A bony exostosis without a cartilage cap may represent a developmental anomaly, posttraumatic reactive bone, or a fully ossified osteochondroma [10–12, 19]. Osteoma corresponds to a dense, compact bone growth, often asymptomatic [18]. Chondrosarcoma is a rare malignant transformation of a preexisting cartilage lesion and should be suspected in cases of rapid growth, pain, or cartilage cap thickening exceeding 2 cm in adults. Omovertebral bone is a congenital bony, cartilaginous, or fibrous bridge between the scapula and cervical spine, usually associated with Sprengel′s deformity [14, 15, 25–28].

Given the clinical, imaging, and histopathological findings, our case is most consistent with a benign exostosis of the scapula. Surgical excision led to complete symptom resolution, in line with previous reports on the treatment of symptomatic scapular bony lesions [1, 15, 18, 19, 27, 29–35].

**Figure 6:**
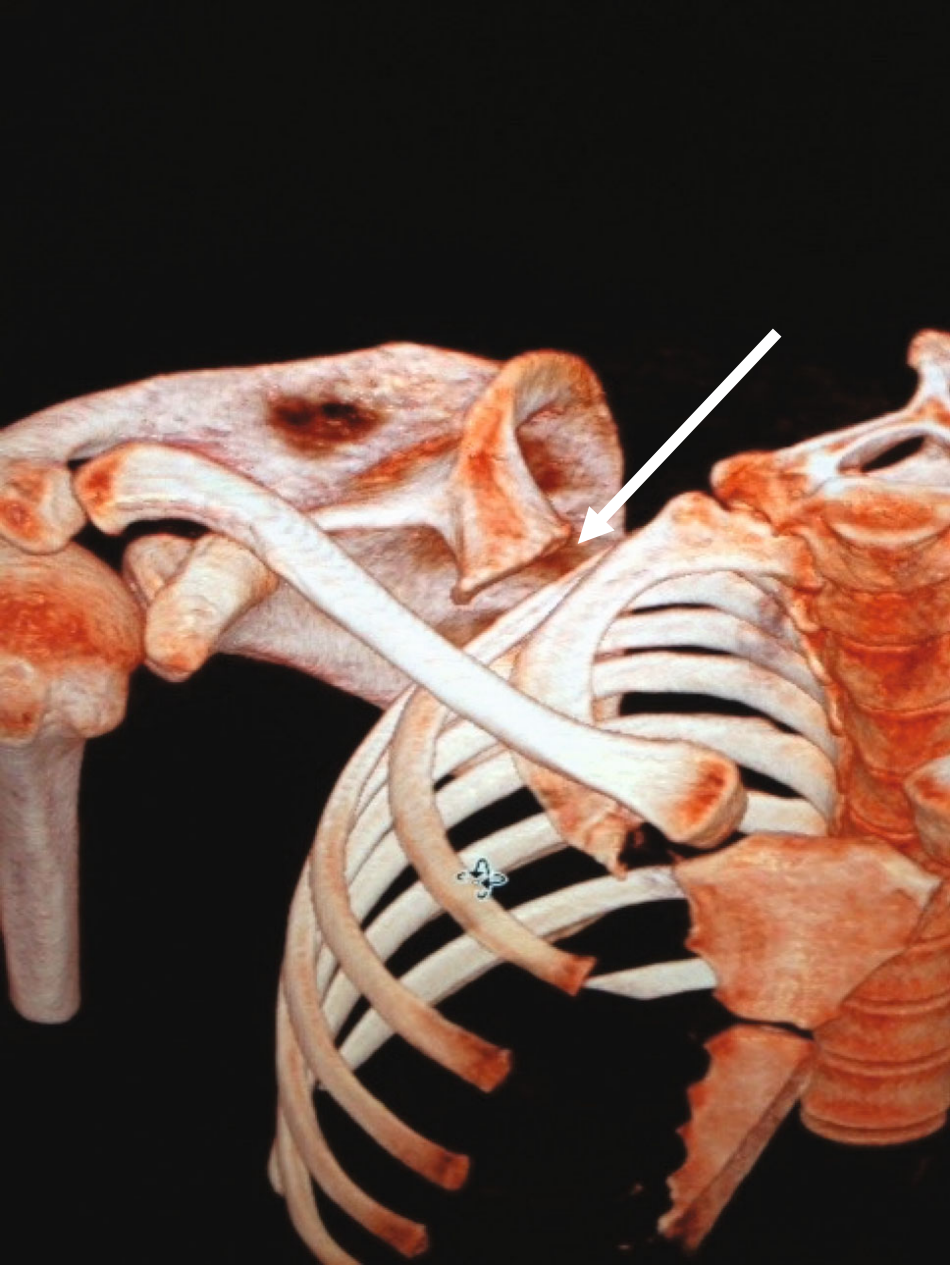
3D reconstruction CT scan of the right shoulder reveals an articulation between the second glenoid and the body of the second rib (white arrow). Reproduced from http://www.BeeMed.com, with permission.

**Figure 7:**
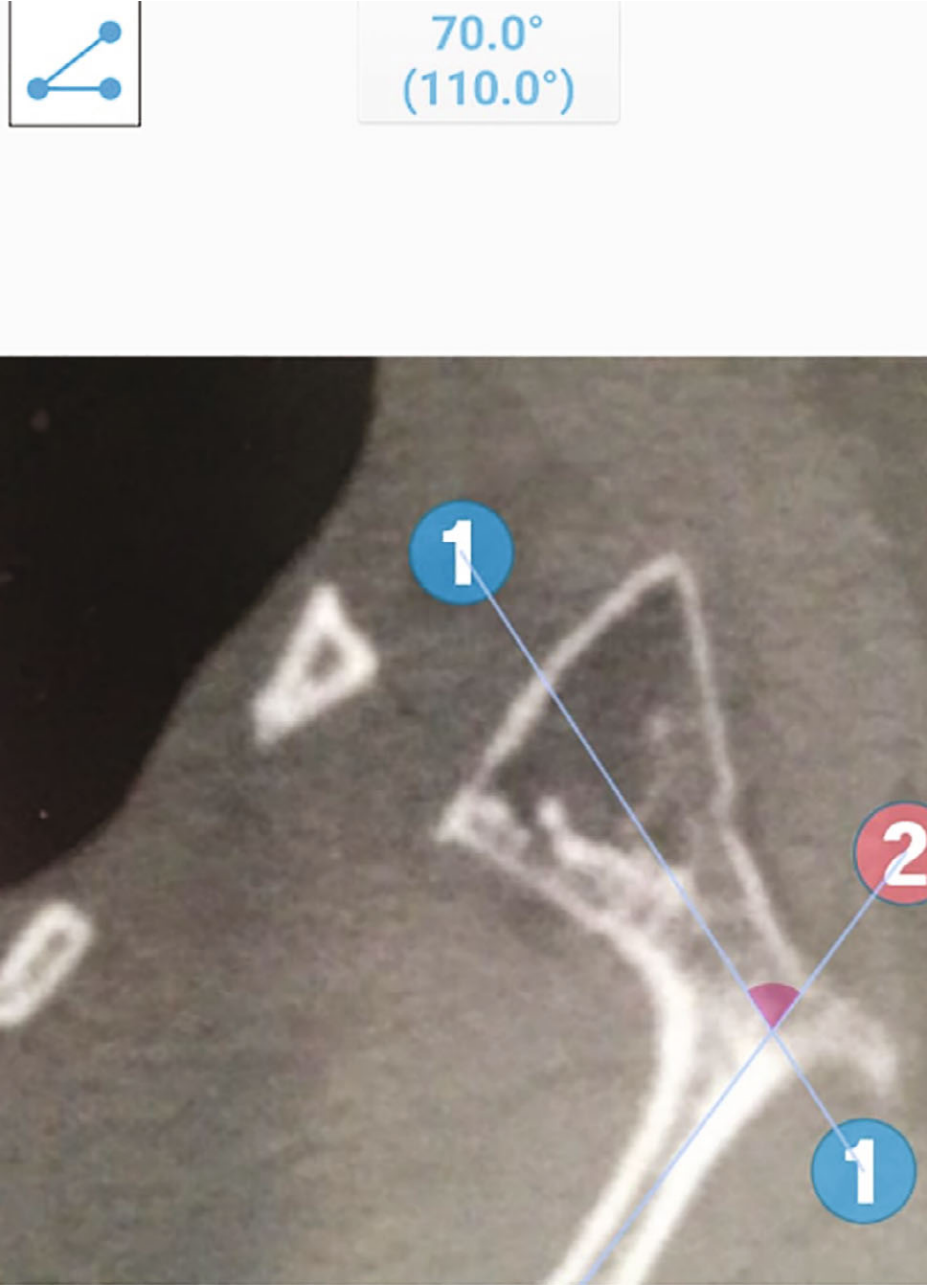
CT scan axial view of the right shoulder displays anteversion of the second glenoid of nearly 70°. Reproduced from http://www.BeeMed.com, with permission.

In the present case, the bony mass is better described as a second glenoid with a coracoid‐like process and a spine at its base (Figure 6). This pseudo‐glenoid articulates with the body of the second rib at its prominent tubercle to form a pseudo‐glenocostal joint. The anteversion of the second glenoid was approximately 70° (Figure 7). In most of the reported cases, surgical excision relieved pain and noise [29–31, 36]. Several authors contend that most cases of snapping scapula have no radiographically demonstrable bone abnormalities [1, 22, 23]. Milch demonstrated that surrounding musculature is involved in many cases, reporting a marked thickening and bursal formation of the subscapularis muscle beneath a portion of the excised scapula [1].

Although the scapula ossifies from multiple centers during development, the available clinical and histopathological findings do not provide definitive evidence for a congenital origin of the lesion. An alternative explanation could be that the bony projection represents an acquired process, such as a reactive exostosis or a long‐standing osteochondroma, that has undergone morphological changes over time due to repetitive scapulothoracic motion. Such mechanical remodeling might explain its shape and apparent contact with the second rib, but this interpretation remains speculative and would require further confirmation from additional imaging or longitudinal data [37].

## 4. Conclusion

Bone masses arising from the scapula differ according to the site of origin; time of presentation; relation to cervical or thoracic vertebrae, clavicle, and rib cage; and the morphology of the mass. We describe a unique bony projection in an adult patient, presenting at 16 years of age, arising from the superior border of the scapula and resembling a second glenoid articulating with the body of the second rib. This condition was surgically treated by resection, resulting in complete cosmetic and functional improvement.

## Consent

Written informed consent was obtained from the patient for publication of this case report and accompanying images.

## Conflicts of Interest

Alaa Elsenbsy and Jeanni Zbinden declare no conflicts of interest. Alexandre Lädermann is a paid consultant for Arthrex, Stryker, Medacta, and Enovis. He received royalties from Stryker and Medacta. He is the (co‐)founder of FORE, Med4Cast, BeeMed, and The Hive Musculoskeletal Journal (THMS). He owns stock options in Follow Health. He is on the board of the French Arthroscopic Society. Michael Simoni is a consultant for Exactech.

## Funding

This study was supported by FORE (Foundation for Research and Teaching in Orthopedics, Sports Medicine, Trauma, and Imaging in the Musculoskeletal System), Grant # 2024‐5.

## Data Availability

All anonymized data are available from the corresponding author upon reasonable request.
